# Survival benefit stratification of partial nephrectomy versus non‐surgical treatment in elderly patients with T1a renal cell carcinoma

**DOI:** 10.1002/cam4.5580

**Published:** 2023-01-11

**Authors:** Yaxiong Tang, Kan Wu, Xu Hu, Yang Liu, Weixiao Yang, Xiang Li

**Affiliations:** ^1^ West China School of Medicine, West China Hospital Sichuan University Chengdu China

**Keywords:** elderly population, non‐surgical treatment, overall survival, partial nephrectomy, renal cell carcinoma

## Abstract

**Background:**

Renal cell carcinoma (RCC) of stage T1a has been proven to be of low‐grade malignancy and mostly affects elderly individuals with relatively limited life expectancy. However, research on the survival benefit of surgery relative to non‐surgical treatment (NST) is limited. The aim of the study was to investigate the survival difference between partial nephrectomy (PN) and NST and to establish a benefit stratification model for elderly patients (≥70 years) diagnosed with T1a RCC.

**Patients and Methods:**

Patients diagnosed with non‐metastatic T1a RCC who received PN or NST were identified from the SEER database during 2004–2015. Before survival analysis, propensity score matching (PSM) was performed. Overall survival (OS) was estimated by the Kaplan–Meier method, and subgroup analyses were used to identify favorable factors of PN. Independent factors of survival were recognized by multivariate Cox regression analysis.

**Results:**

Patients diagnosed with non‐metastatic T1a RCC who received PN or NST were identified from the SEER database during 2004–2015. Before survival analysis, propensity score matching (PSM) was performed. Overall survival (OS) was estimated by the Kaplan–Meier method, and subgroup analyses were used to identify favorable factors of PN. Independent factors of survival were recognized by multivariate Cox regression analysis.

**Conclusions:**

Our findings suggest that the survival benefit of PN could be stratified based on the clinical characteristics in patients with stage T1a RCC aged 70 years or older, which may help physicians and patients optimize clinical decisions.

## Graphical abstract

The overall survival benefit of partial nephrectomy over non‐surgical treatment could be stratified based on the patient's clinical characteristics in patients with stage T1a RCC aged 70 years or older.

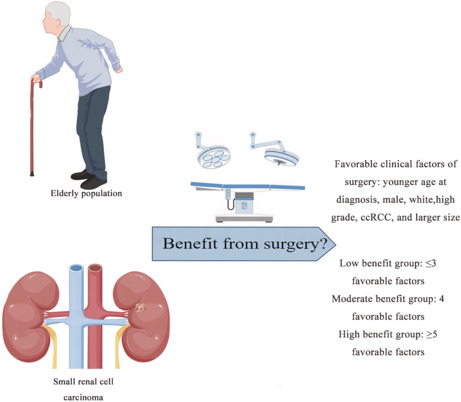

## 1 INTRODUCTION

Renal cell carcinoma (RCC) is one of the most common malignancies, accounting for 2.2% of all new cancer cases and 1.8% of cancer deaths worldwide in 2020.^1^ Due to the widespread use of axial imaging, the incidence of kidney tumors is increasing at a rate of 1% per year and occurs mainly in populations aged 75 years or older.^2^, ^3^ Small renal mass (SRM), which is defined as tumor ≤4 cm, accounts for approximately 40% of all incidental renal masses, of which approximately 80% are RCC.^4^, ^5^ Recommended by the American Urological Association (AUA), European Association of Urology (EAU), and National Comprehensive Cancer Network (NCCN), partial nephrectomy (PN) is currently the standard treatment for patients with stage T1a RCC, reserving active surveillance (AS) to patients with comorbidities.^3^, ^6^, ^7^ With the fact that renal tumors mainly affect elderly individuals with relatively limited life expectancy and SRMs are mostly low Fuhrman grade (I–II), slow growing and with a low probability of metastasis,^7^, ^8^, ^9^, ^10^, ^11^, ^12^, ^13^ the complications and oncological benefits caused by surgery should be carefully considered in elderly patients diagnosed with SRM. Moreover, due to the advanced age at diagnosis, patients with AS may become unfit for surgery when the disease progresses to the point where surgery is required because of worsening performance status during follow‐up. Therefore, it is necessary to identify which populations would benefit more from surgery and to prioritize surgery for these populations. However, research comparing the effects of surgery and non‐surgical treatment (NST) on survival is limited and lacks a benefit stratification model for such patients.^14^

Based on the NCI's Surveillance, Epidemiology, and End Results (SEER) database, we aimed to investigate the overall survival (OS) difference between PN and NST and to establish a benefit stratification model for elderly patients diagnosed with stage T1a RCC.

## 2 PATIENTS AND METHODS

### 2.1 Study population

The NCI's SEER database, which is a national registry that records approximately 28% of cancer‐related information from the United States, was used to obtain information on patients who met our criteria.

**FIGURE 1:**
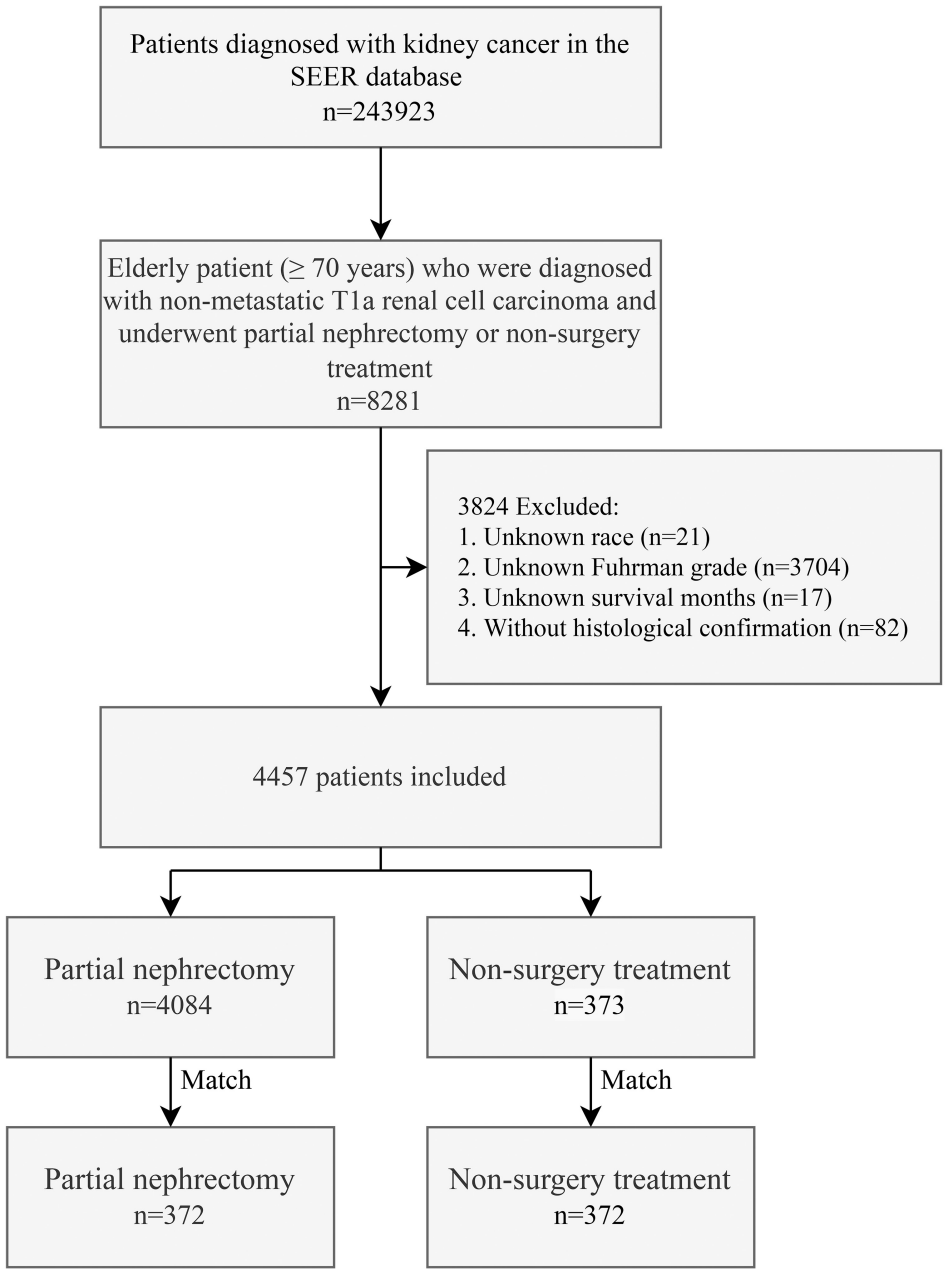
CONSORT diagram.

The inclusion criteria were as follows: (1) patients diagnosed with stage T1a RCC (primary site C64.9), without lymph node and distant metastasis, and aged 70 years or older; and (2) patients who received NST (code 00) or PN (code 30). Patients with unknown race, Fuhrman grade, survival time, and no histological confirmation were excluded from our study. The screening process is as shown in Figure 1.

### 2.2 Clinical covariates

Demographic variables included the patient's age at diagnosis, sex, race, and year of diagnosis. Oncologic variables included the Fuhrman grade, tumor size, histological type, treatment modality, survival status, and survival time. Age at diagnosis was divided into 70–79 and ≥80 years. Race was defined as white and others. Fuhrman grade was categorized as low grade (LG, Fuhrman grade I–II) and high grade (HG, Fuhrman grade III–IV). Tumor size was divided into 0.1–2.0 cm and 2.1–4.0 cm. Histology was categorized into clear cell RCC (ccRCC) and non‐clear cell RCC (non‐ccRCC). Treatment modalities included NST and PN.

### 2.3 Statistical analysis

Patients who met the inclusion criteria were divided into the PN group and NST group according to the treatment received. Propensity score matching (PSM) was used to balance clinical variables between the two groups. The clinical characteristics of the two groups of patients before and after PSM were described as categorical or count variables and compared with the chi‐square or Mann–Whitney *U* test as appropriate. After PSM, the OS of the two groups was estimated by the K‐M curve and compared with the log‐rank test. We next performed subgroup survival analysis based on prespecified clinical variables. Finally, univariate and multivariate Cox regression analyses were performed to identify independent factors for survival.

### 2.4 Survival benefit stratification

Based on the results of the subgroup analysis, variables with lower hazard ratio (HR) were defined as favorable factors of PN in each subgroup, including younger age at diagnosis (70–79 years), male sex, white race, HG, ccRCC histology, and larger size (2.1–4.0 cm). Based on the number of favorable factors of PN, patients were divided into 3 predictive subgroups: low benefit group (≤3 favorable factors), moderate benefit group (4 favorable factors), and high benefit group (≥5 favorable factors). With the aim of investigating the overall survival benefit of PN relative to NST in each group, the K–M curve was used to estimate OS with the log‐rank test for comparison. All tests were two‐sided, and *p* < 0.05 was considered statistically significant. Statistical analyses were performed by SPSS (version 25.0) and R software (version 4.2.1).

## 3 RESULTS

### 3.1 Population characteristics

A total of 4457 patients were included in the study from the SEER database between 2004 and 2015, of which 4084 patients (91.6%) received PN and 373 patients (8.4%) received NST. The median age of all enrolled patients, patients who received PN, and patients who received NST were 74 (interquartile range, 71–77), 74 (interquartile range, 71–77), and 75 (interquartile range, 71–80) years, respectively. Patients who received NST were older than those who received PN (*p* < 0.001). The majority of patients were male (60.1%), white (84.6%), aged 70–79 years (83.7%), and presented with ccRCC (59.7%), LG (77.5%), and 2.1–4 cm (70.0%). Before PSM, age, Fuhrman grade, and tumor size differed in patients who received NST and PN (all *p* ≤ 0.001). After PSM, 372 patients each received NST and PN, and there was no difference in clinical characteristics between the two groups (all *p* > 0.7). The clinical characteristics of the population before and after PSM are shown in Table 1.

**TABLE 1:** Demographic and oncologic characteristics of included patients

Variables	Total (*n* = 4457)	Before PSM			After PSM		
		NST (*n* = 373)	PN (*n* = 4084)	*p*	NST (*n* = 372)	PN (*n* = 372)	*p*
Age, median (IQR)	74 (71, 77)	75 (71, 80)	74 (71, 77)	<0.001	75 (71, 80)	75 (71, 80)	0.848
Age, years				<0.001			1
70–79	3730 (83.7%)	212 (56.8%)	3518 (86.1%)		212 (57.0%)	212 (57.0%)	
≥80	727 (16.3%)	161 (43.2%)	566 (13.9%)		160 (43.0%)	160 (43.0%)	
Gender				0.318			0.882
Male	2677 (60.1%)	215 (57.6%)	2462 (60.3%)		215 (57.8%)	213 (57.3%)	
Female	1780 (39.9%)	158 (42.4%)	1622 (39.7%)		157 (42.2%)	159 (42.7%)	
Race				0.417			0.746
White	3771 (84.6%)	321 (86.1%)	3450 (84.5%)		321 (86.3%)	324 (87.1%)	
Others	686 (15.4%)	52 (13.9%)	634 (15.5%)		51 (13.7%)	48 (12.9%)	
Year at diagnosis				0.118			0.938
2004–2009	1636 (36.7%)	123 (33.0%)	1513 (37.0%)		122 (32.8%)	123 (33.1%)	
2010–2015	2821 (63.3%)	250 (67.0%)	2571 (63.0%)		250 (67.2%)	249 (66.9%)	
Histology				0.785			0.764
ccRCC	2659 (59.7%)	225 (60.3%)	2434 (59.6%)		224 (60.2%)	228 (61.3%)	
Non‐ccRCC	1798 (40.3%)	148 (39.7%)	1650 (40.4%)		148 (39.8%)	144 (38.7%)	
Grade				0.001			0.759
Low grade (I–II)	3453 (77.5%)	315 (84.5%)	3138 (76.8%)		314 (84.4%)	317 (85.2%)	
High grade (III–IV)	1004 (22.5%)	58 (15.5%)	946 (23.2%)		58 (15.6%)	55 (14.8%)	
Size, cm				<0.001			0.927
0.1–2.0	1336 (30.0%)	75 (20.1%)	1261 (30.9%)		74 (19.9%)	73 (19.6%)	
2.1–4.0	3121 (70.0%)	298 (79.9%)	2823 (69.1%)		298 (80.1%)	299 (80.4%)	

Abbreviations: ccRCC, clear cell renal cell carcinoma; IQR, interquartile range; NST, non‐surgical treatment; PN, partial nephrectomy; PSM, propensity score matching.

### 3.2 Overall survival

**FIGURE 2:**
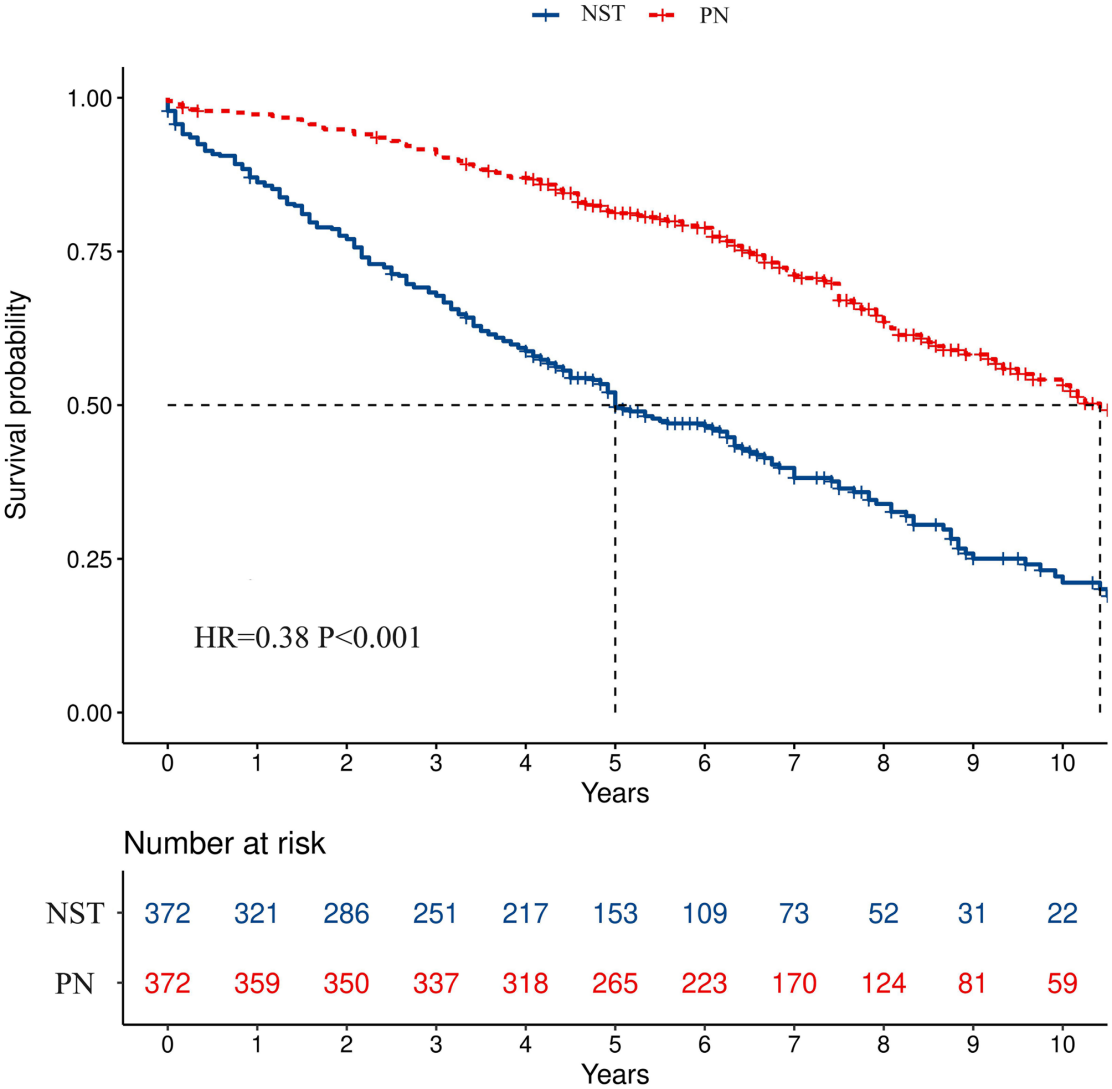
Kaplan–Meier survival curves in the complete cohort.

**FIGURE 3:**
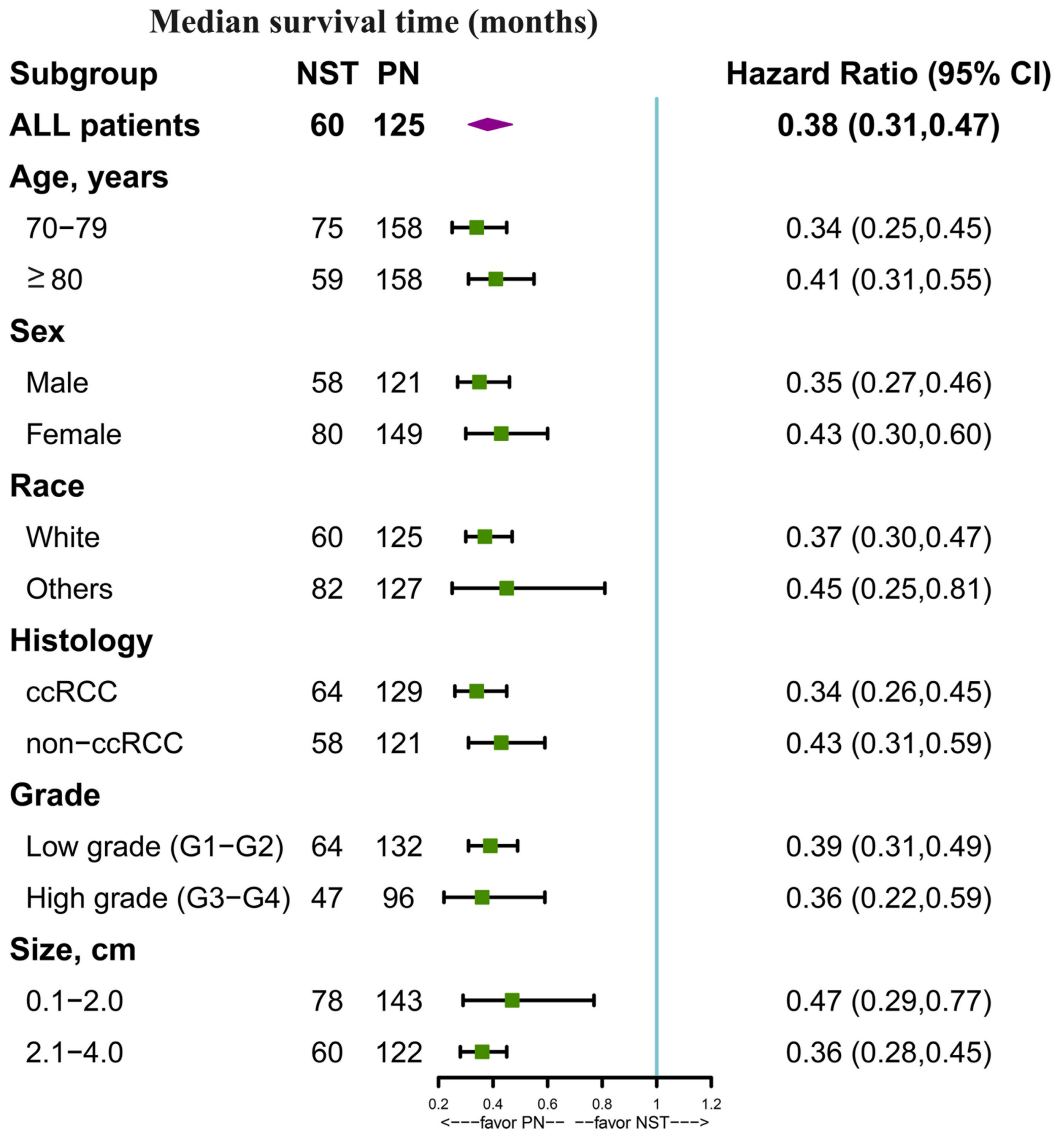
Effect of PN in all prespecified subgroups.

After PSM, survival analysis was performed. Compared with NST, PN resulted in better OS in all populations (HR = 0.38, *p* < 0.001, Figure 2). Similar results were found in all subgroups (all HR < 1, *p* < 0.05), however, the magnitude of survival benefit varied, with PN achieving greater benefit in patients aged 70–79 years, males, white, and patients with HG, ccRCC, and larger tumors (Figure 3).

### 3.3 Survival benefit stratification

**FIGURE 4:**
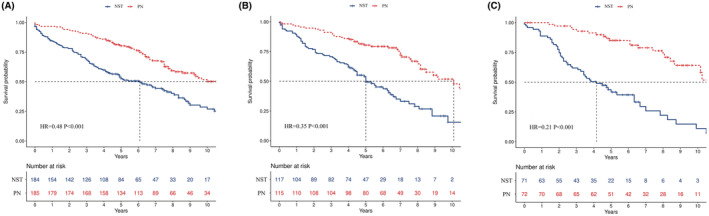
Kaplan–Meier survival curves showing the survival difference between NST and PN patients in the low (A), moderate (B), and high (C) survival benefit cohorts.

Based on the results of the subgroup analysis, the clinical variables with greater benefit in the subgroup analysis were defined as favorable factors of PN, which included younger age at diagnosis (70–79 years), male sex, white race, HG, ccRCC and larger tumor size (2.1–4 cm). According to the number of favorable factors of PN, we divided the patients into 3 groups of low benefit (≤3 favorable factors), moderate benefit (4 favorable factors), and high benefit (≥5 favorable factors) and found that compared with NST, with the increase in the favorable factors, the greater the survival benefit (low benefit, HR = 0.48, 95% CI, 0.36–0.64, *p* < 0.001, Figure 4A; moderate benefit, HR = 0.35, 95% CI, 0.24–0.52, *p* < 0.001, Figure 4B; high benefit, HR = 0.21, 95% CI, 0.12–0.36, *p* < 0.001, Figure 4C).

### 3.4 Cox regression

Age, sex, tumor size, Fuhrman grade, and treatment modality were identified as potential independent predictors in the univariate Cox analysis and were then included in the multivariate Cox analysis (all *p* < 0.05). In multivariate Cox analysis, ≥80 years (HR = 1.49; 95% CI, 1.22–1.83; *p* < 0.001), HG (HR = 1.33; 95% CI, 1.03–1.73; *p* = 0.032), and larger tumor size (HR = 1.31; 95% CI, 1.01–1.70; *p* = 0.047) were identified as independent risk factors, and female sex (HR = 0.73; 95% CI, 0.59–0.90; *p* = 0.003) and PN (HR = 0.37; 95% CI, 0.30–0.46; *p* < 0.001) were identified as independent protective factors for OS. The results of the Cox regression are shown in Table 2.

**TABLE 2:** Univariate and multivariate Cox regression analyses for OS in elderly patients diagnosed with T1a RCC

Variables	Univariable		Multivariable	
	HR (95%CI)	*p*	HR (95%CI)	*p*
Age, years				
70–79	Ref		Ref	
≥ 80	1.48 (1.21, 1.81)	<0.001	1.49 (1.22, 1.83)	<0.001
Gender				
Male	Ref		Ref	
Female	0.76 (0.62, 0.93)	0.009	0.73 (0.59, 0.90)	0.003
Race				
White				
Others	0.89 (0.66, 1.21)	0.456		
Histology				
ccRCC	Ref			
Non‐ccRCC	1.13 (0.92, 1.38)	0.241		
Grade				
Low grade (I–II)	Ref		Ref	
High grade (III–IV)	1.46 (1.12, 1.89)	0.005	1.33 (1.03, 1.73)	0.032
Size, cm				
0.1–2.0	Ref		Re	
2.1–4.0	1.34 (1.03, 1.74)	0.029	1.31 (1.00, 1.70)	0.047
Treatment				
NST	Ref		Ref	
PN	0.38 (0.31, 0.47)	<0.001	0.37 (0.30, 0.46)	<0.001

Abbreviations: ccRCC, clear cell renal cell carcinoma; HR, hazard ratio; NST, non‐surgical treatment; PN, partial nephrectomy.

## 4 DISCUSSION

Using a large national registry, we found that PN resulted in better OS than NST, and the magnitude of the survival benefit was related to the clinical characteristics of the population in elderly patients diagnosed with stage T1a RCC.

In the current era, RCC of stage T1a is considered to have low malignant potential. In a SEER database‐based study of 13,364 patients, it was reported that only 14.5% of patients had Fuhrman grade III–IV ccRCC or papillary RCC among patients who were diagnosed with T1a RCC and underwent nephrectomy, and the proportion of synchronous metastases was almost zero even in patients with Fuhrman grade III–IV.^11^ In 3 prospective clinical studies, the average growth rate of SRM was reported to be 0.13, 0.25 and 0.11 cm per year.^8^, ^9^, ^10^ However, risk may be individual. In a study that included 136 patients with histologically confirmed RCC, ccRCC was found to grow faster than papillary type 1 RCC, and all 6 patients with metastases were histologically ccRCC during follow‐up.^15^ As our research found, patients with high‐grade, larger tumors and ccRCC, who are considered to have greater risk of progression, benefited more from PN. These findings indicated the importance of biopsy to guide assessment of patient risk and decision‐making on monitoring and treatment.

Due to the low malignant potential, major guidelines refer to AS as a treatment option (not preferred), especially in the elderly and with comorbidities,^3^, ^6^, ^7^ and current studies have focused on the survival outcome between AS and surgery. In a prospective study that included 497 patients with SRM, AS demonstrated non‐inferior OS and cancer‐specific‐survival (CSS) relative to PN.^10^ AS also resulted in non‐inferior cancer‐specific mrtality compared with surgery for the elderly population with localized RCC in several population‐based studies.^16^, ^17^, ^18^ Although studies have shown that AS is appropriate for selected patients and the fact that elderly patients with T1a RCC have mostly low‐grade tumors and limited life expectancy, few studies have focused on the necessity and efficacy of surgery for such populations.^14^ After all, AS requires regular follow‐up, which can be a burden for part of the population. In addition, patients who received AS were usually older and more frail,^19^, ^20^, ^21^ and they may become ineligible for surgery due to new‐onset disease or worsening of other preexisting conditions during follow‐up. Based on these concerns, it is vital to identify patients who would benefit more from surgery and who may become ineligible for surgery due to worsening performance status during follow‐up, thus providing surgery rather than AS as the first choice for such patients.

It was reported that nephrectomy resulted in better survival than NST in a previous study without age limitation, which was similar to our finding.^14^ Based on the SEER database, we found that PN resulted in better OS than NST and established a survival benefit stratification model based on the results of prespecified subgroup analyses. The survival benefit of PN relative to NST was present in every stratification and was more pronounced with increasing favorable factors of PN. For patients with greater survival benefit and who may not undergo surgery due to worsening performance status during follow‐up, it may be better to have surgery as the first choice.

Unsurprisingly, advanced age was identified as an independent risk factor for survival in the multivariate Cox regression analysis in our study. However, it is worth noting that this does not mean that older patients will benefit more from PN. In such a population with a relatively limited life expectancy, the relatively young will definitely have a better survival regardless of disease. Therefore, it is the subgroup analysis that can be more representative of the degree of survival benefit caused by PN. However, the survival benefit may be overestimated, it is evident that surgeons prefer to choose more fit candidates for PN and reserve NST for the fragile. In a study that included patients with stage T1 RCC, patients with AS were older and had more comorbidities than patients treated with surgery.^19^ Likewise, a study based on the prospective, multi‐institutional Delayed Intervention and Surveillance for Small Renal Masses (DISSRM) Registry found that patients who received immediate intervention had better baseline quality of life than those who received AS, especially in domains that reflected physical health.^20^ However, with the fact that potential bias existed in each stratification, we believe it is the magnitude of benefit, not the benefit stratification, that is affected.

There are several limitations of our study. First, although based on a large registry, the sample size of NST was limited and lacked validation of external cohorts, which may affect our conclusions to some extent. Second, although PSM was performed to balance clinical variables, some important patient information, such as performance status and comorbidities, was not available in the SEER database, so potential bias may exist. However, we believe that stratification is unaffected, as potential bias should be present in all subgroups. Therefore, the most important significance of our study is to reveal how population characteristics and tumor characteristics affect the oncology outcome in such patients, rather than the absolute value of survival benefit caused by PN versus NST. Third, due to the limitation of sample size, non‐ccRCC, a group of heterogeneous tumors, were unable to be classified more specifically. Finally, some other important outcomes were not available in the SEER database, such as progression‐free survival and patient quality of life. Large, prospective studies are needed.

## 5 CONCLUSION

Based on the SEER database, we found that PN resulted in an OS benefit over NST, and the benefit could be stratified based on the patient's clinical characteristics in patients with stage T1a RCC aged 70 years or older. This can help physicians and patients optimize clinical decisions.

## AUTHOR CONTRIBUTIONS

**Yaxiong Tang:** Data curation (equal); formal analysis (equal); methodology (equal); software (equal); writing – original draft (equal); writing – review and editing (equal). **Kan Wu:** Data curation (equal); formal analysis (equal); methodology (equal). **Xu Hu:** Data curation (equal); methodology (equal); project administration (equal); validation (equal). **Yang Liu:** Data curation (equal); formal analysis (equal); software (equal); validation (equal). **Weixiao Yang:** Funding acquisition (equal); investigation (equal); writing – original draft (equal); writing – review and editing (equal). **Xiang Li:** Conceptualization (equal); funding acquisition (equal); project administration (equal); supervision (equal); validation (equal); writing – review and editing (equal).

## FUNDING INFORMATION

This work was supported by the Sichuan Science and Technology Program (Reference Number: 2022YFS0133).

## CONFLICT OF INTEREST

The authors declare that the research was conducted in the absence of any commercial or financial relationships that could be construed as a potential conflict of interest.

## ETHICAL STATEMENT

The study was derived from a public database, and sensitive patient information has been obscured, so the study was exempt from ethical approval and informed consent from the Institutional Review Board.

## Data Availability

The data that support the findings of this study are openly available; for more information, please visit seer.cancer.gov.
